# Association between postterm birth and adverse growth outcomes in children aged 3–6 years: A national retrospective cohort study

**DOI:** 10.1111/ppe.13122

**Published:** 2024-09-19

**Authors:** Marini Ahmad Suhaimi, Yingyan Zheng, Haizhen You, Yuantao Su, Gareth J. Williams, Manish Prasad Gupta, Wenchong Du, Jing Hua

**Affiliations:** ^1^ Shanghai Key Laboratory of Maternal Fetal Medicine, Shanghai Institute of Maternal‐Fetal Medicine and Gynecologic Oncology Shanghai First Maternity and Infant Hospital, School of Medicine, Tongji University Shanghai China; ^2^ NTU Psychology Nottingham Trent University Nottingham UK; ^3^ Department of Scientific Research, National Children's Medical Center Children's Hospital of Fudan University Shanghai China; ^4^ School of Social Sciences Nottingham Trent University Nottingham UK

**Keywords:** body mass index, childhood obesity, gestational age, overweight, postterm birth, thinness

## Abstract

**Background:**

Gestational age significantly influences children's growth and development. Yet, the effect of postterm birth (gestation beyond 42 weeks) on children's growth outcomes remains underexplored.

**Objectives:**

This study aimed to assess the impact of postterm birth on adverse growth outcomes in children using a nationally representative sample from China.

**Methods:**

A retrospective cohort study was conducted in China from 1 April 2018, to 31 December 2019. The final analysis included 141,002 children aged 3–6 years from 551 cities. Postterm birth was defined as children with postterm birth at a gestational age of 42 weeks or more. Obesity, overweight and thinness were assessed using body mass index‐for‐age (BMI‐for‐age) *z*‐scores, based on the World Health Organization (WHO) Child Growth Standards. Generalised additive models were employed to investigate the non‐linear relationship between maternal gestational age and BMI‐for‐age *z* scores. Poisson regression models and subgroup analyses with forest plots were performed to examine the associations between postterm birth and the risks of obesity, overweight and thinness in children.

**Results:**

We included 141,002 mother–child pairs, of whom 7314 (5.2%) children were classified as postterm births. There exists a non‐linear relationship between gestational age and BMI‐for‐age *z* scores. Children born postterm exhibited a 46% increased risk of obesity, a 27% increased risk of combined overweight/obesity and a 13% increased risk of thinness. Similar associations were observed in most cases when further sensitivity and subgroup analysis were conducted.

**Conclusions:**

Postterm birth was associated with elevated risks of obesity, overweight and thinness in children aged 3–6 years, independent of sex. These findings underscore the importance of further research across diverse populations to understand the implications of postterm births on child health outcomes.

**Synopsis**

**Study question**

How does postterm birth (gestation beyond 42 weeks) impact adverse growth outcomes such as obesity, overweight and thinness in children?

**What is already known**

Gestational age significantly influences children's growth and development, yet the effects of postterm birth on these outcomes remain underexplored.

**What this study adds**

This study utilises a large, nationally representative sample from China to establish that postterm birth was associated with a higher risk of obesity, overweight and thinness in children. It highlights the need for targeted research and interventions to address the negative consequences of extended gestational age.

## 1 INTRODUCTION

Gestational age is known to be associated with children's long‐term health outcomes.^1^, ^2^ While numerous studies have reported an increased risk of developmental and health issues in children born preterm, limited research has focused on postterm birth. Postterm birth is defined as birth that takes place after 42 completed weeks of gestation.^3^ Globally, the incidence of postterm births varies, with rates reaching up to 10%.^4^ Postterm births lead to an increased risk of maternal morbidity,^3^ caesarean section^5^ and haemorrhage,^6^ Moreover, postterm births are also associated with adverse outcomes such as newborn birth injuries,^7^ an increased risk of stillbirth,^4^ neonatal and infant death.^8^, ^9^

Research indicates that postterm births are associated with negative developmental outcomes, such as impaired neurodevelopment marked by cognitive and behavioural delays, as well as motor developmental delays in fine and gross motor skills.^10^, ^11^, ^12^, ^13^, ^14^, ^15^, ^16^, ^17^ Additionally, there is emerging evidence of a higher incidence of neurodevelopmental disorders, including autism spectrum disorder (ASD) and attention‐deficit/hyperactivity disorder (ADHD), among this population.^18^, ^19^, ^20^, ^21^ Despite these findings, to date, only three studies have provided evidence on the association between postterm birth and subsequent growth of children, yielding inconsistent results. Specifically, one study indicates an increased risk of obesity in postterm boys but not girls,^22^ another was limited to female participants only,^23^ while the third suggest a heightened risk of obesity or even a reduced risk across both sexes in different populations.^24^ These studies often face limitations such as small sample sizes, geographic restrictions and inadequate adjustment for confounding variables, which further complicate the ability to draw definitive conclusions.^22^, ^23^, ^24^

Given the limited number of studies available and the discrepancies in their findings, drawing definitive conclusions regarding the association between postterm birth and subsequent growth outcomes remains challenging. Therefore, the current study aimed to involve a larger and more diverse study population and rigorous adjustment for confounding factors, to gain a comprehensive understanding of the potential effects of postterm birth on the risk of adverse growth outcomes in preschool children. In the current study, we used a retrospective cohort design to examine the association between postterm birth and risk of adverse growth outcomes including obesity, overweight and thinness with a national sample of Chinese children born after cessation of the one‐child policy. We hypothesised that a higher risk of obesity and thinness may occur in postterm preschoolers.

## 2 METHODS

### 2.1 Study design and participants

The study sample was from the Chinese National Cohort of Motor Development (CNCMD).^15^, ^17^, ^25^ In order to ensure that the participants in the current study were representative of the Chinese population, we employed a stratified cluster sampling approach in the current study. The stratification variables included age, sex, region and socioeconomic status (SES), which were based on the Chinese 2018–2019 National Census. The maternity and children's health centre in each city was invited to involve their local kindergartens to participate in the study. Class teachers were responsible for distributing the notification to parents to complete the online questionnaire. Mothers' identifications (IDs) were collected and used to retrospectively link mothers' maternity clinical records with their children's records.

**FIGURE 1:**
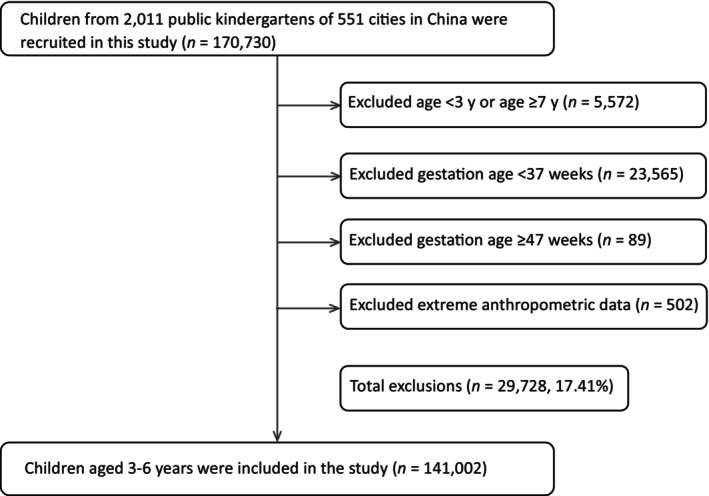
Flowchart of the study sample.

The study only included mainstream schools and nurseries, excluding children who were mandated by local legislation to attend special education schools or nurseries due to significant visual, hearing or intellectual impairments or other serious developmental disorders. From 1st April, 2018 to 31st December, 2019, a total of 170,730 children from 2011 public kindergartens of 551 cities in China were recruited for the study. Children with any of the following conditions were excluded from the study: (1) age <3 years or age ≥7 years; (2) gestation age <37 weeks; (3) gestation age ≥47 weeks; (4) extreme anthropometric data. Finally, 141,002 children aged 3–6 years were included in the study (Figure 1).

### 2.2 Outcome

The height and weight of the children were initially reported by parents and subsequently verified through physical examination records obtained from the nurseries. Our analysis utilised the body mass index‐for‐age BMI‐for‐age *z*‐scores to measure growth outcomes, a standard method that assesses relative weight status based on the ratio of weight to height squared (BMI = weight (kg)/height (m)^2^) and adjusts for child age and sex. According to the WHO Child Growth Standards,^26^ for children aged 3–4 years, a BMI‐for‐age *z*‐score between +2 and +3 indicated overweight and a *z*‐score above +3 indicated obesity. For children aged 5–6 years, overweight was defined as a BMI‐for‐age *z*‐score between +1 and +2 and obesity as a *z*‐score above +2. Thinness was identified by a BMI‐for‐age *z*‐score below −2, adhering to the study‐specific criteria.^27^

### 2.3 Exposures

Gestational age at birth was determined using the mother's medical records, which included information from ultrasound examinations and the date of the previous menstrual cycle. The exposed group was defined as children with postterm birth at a gestational age of 42 weeks or more, in line with previous research.^3^ The unexposed group was defined as children born at term, with gestational ages between 37 and <42 weeks.

### 2.4 Covariates

In this study, we investigated the association of gestational age with adverse growth outcomes in children, taking into account various child and family characteristics as variables. These variables were selected based on the findings from our literature review. Table 1 provides a comprehensive list of the variables considered in this analysis: (1) Child characteristics included the child's age, sex, eyesight, right‐handedness, breast feeding longer than 6 months, birth weight, delivery mode, parity, neonatal intensive care unit (NICU) admission, other developmental disorders (attention‐deficit/hyperactivity disorder, learning disorders and autism spectrum disorder, etc.), and psychiatric medication. (2) Family characteristics included mother's age, father's age, mother's BMI, father's BMI, higher education of mother or father, occupation, family structure, maternal complications during pregnancy. The term *breast feeding longer than 6 months* was used to describe whether an infant was breastfed for more than 6 months. The term *higher educatio*n refers to tertiary education that results in the awarding of an academic degree, including associate degrees, bachelor's degrees, master's degrees and doctoral degrees. Family structures included extended families, nuclear families and single‐parent families. In the context of this study, an *extended family* refers to a child living with both parents and grandparents, which is a common family structure in China; the term *nuclear family* refers to a child who lives with only their parents; the term *single‐parent family* means that the child lives with only one of their parents. Maternal complications during pregnancy were defined using the International Classification of Diseases‐Revision 10 (ICD‐10). This classification encompassed conditions such as intrauterine distress, foetal asphyxia, risk of miscarriage, vaginal bleeding during pregnancy and the usage of antibiotics or fertility medications. These factors were accounted for to better understand their potential influence on the relationship between gestational age and adverse growth outcomes in children.

**TABLE 1:** Characteristics of the study population.

Characteristics	Total (*n* = 141,002)	Term birth (*n* = 133,688)	Postterm birth (*n* = 7314)
Child age (years)	4.6 (0.9)	4.6 (0.9)	4.7 (0.9)
Child sex, *n* (%)			
Male	74,694 (53.0)	70,956 (53.1)	3738 (51.1)
Female	66,308 (47.0)	62,732 (46.9)	3576 (48.9)
Mother's age (years)	32.1 (4.6)	32.1 (4.6)	31.8 (4.8)
Father's age (years)	33.7 (5.5)	33.8 (5.5)	33.5 (5.6)
Mother's BMI (kg/m^2^)	21.7 (3.2)	21.7 (3.2)	21.9 (3.4)
Father's BMI (kg/m^2^)	23.5 (3.2)	23.5 (3.2)	23.4 (3.2)
Eyesight^a^, *n* (%)			
Normal	127,054 (90.1)	120,399 (90.1)	6655 (91.0)
Abnormal	13,948 (9.9)	13,289 (9.9)	659 (9.0)
Right‐handedness, *n* (%)			
No	9971 (7.1)	9422 (7.0)	549 (7.5)
Yes	131,031 (92.9)	124,266 (93.0)	6765 (92.5)
Breast feeding longer than 6 months^b^, *n* (%)			
No	27,636 (19.6)	26,314 (19.7)	1322 (18.1)
Yes	113,366 (80.4)	107,374 (80.3)	5992 (81.9)
Birth weight (g)	3297.2 (565.5)	3292.3 (564.0)	3385.7 (584.2)
Mode of delivery, *n* (%)			
Vaginal	75,590 (53.6)	71,590 (53.6)	4000 (54.7)
Caesarean	65,412 (46.4)	62,098 (46.4)	3314 (45.3)
NICU admission, *n* (%)			
No	129,090 (91.6)	122,363 (91.5)	6727 (92.0)
Yes	11,912 (8.4)	11,325 (8.5)	587 (8.0)
Other developmental disorders^c^, *n* (%)			
No	140,171 (99.4)	132,907 (99.4)	7264 (99.3)
Yes	831 (0.6)	781 (0.6)	50 (0.7)
Psychiatric medication, *n* (%)			
No	140,050 (99.3)	132,779 (99.3)	7271 (99.4)
Yes	952 (0.7)	909 (0.7)	43 (0.6)
Maternal complications during pregnancy^d^, *n* (%)			
No	134,682 (95.5)	127,580 (95.4)	7102 (97.1)
Yes	6320 (4.5)	6108 (4.6)	212 (2.9)
Parity, *n* (%)			
1	137,915 (97.8)	130,739 (97.8)	7176 (98.1)
≥2	3087 (2.2)	2949 (2.2)	138 (1.9)
Higher education of mother^e^, *n* (%)			
No	65,183 (46.2)	61,114 (45.7)	4069 (55.6)
Yes	75,819 (53.8)	72,574 (54.3)	3245 (44.4)
Higher education of father^e^, *n* (%)			
No	65,937 (46.8)	61,976 (46.4)	3961 (54.2)
Yes	75,065 (53.2)	71,712 (53.6)	3353 (45.8)
Mother's occupation, *n* (%)			
Employed	87,967 (62.4)	83,391 (62.4)	4576 (62.6)
Unemployed	53,035 (37.6)	50,297 (37.6)	2738 (37.4)
Father's occupation, *n* (%)			
Employed	111,559 (79.1)	105,853 (79.2)	5706 (78.0)
Unemployed	29,443 (20.9)	27,835 (20.8)	1608 (22.0)
Family structure, *n* (%)			
Single‐parent families	3185 (2.3)	2941 (2.2)	244 (3.3)
Nuclear families	87,959 (62.4)	83,186 (62.2)	4773 (65.3)
Extended families	49,858 (35.4)	47,561 (35.6)	2297 (31.4)

Abbreviations: BMI, body mass index (calculated as weight in kilograms divided by height in meters squared); NICU, neonatal intensive care unit.

^a^ Eyesight: the power or faculty of seeing.

^b^ Breast feeding longer than 6 months: whether an infant was breastfed for more than 6 months.

^c^ Other developmental disorders: attention‐deficit/hyperactivity disorder, learning disorders and autism spectrum disorder.

^d^ Maternal complications during pregnancy: the presence of one of the following maternal complications during pregnancy: vaginal bleeding, risk of miscarriage, use of antibiotics, use of fertility drugs, intrauterine distress or foetal asphyxia.

^e^ Higher education of mother or father: tertiary education leading to the award of an academic degree, including associate degrees, bachelor's degrees, master's degrees and doctoral degrees.

### 2.5 Statistical analysis

Generalised additive models were used to investigate the non‐linear relationship between maternal gestational age and BMI‐for‐age *z* scores. To assess the relationship between postterm birth and weight status outcomes in children compared to those born at term, we conducted Poisson regression models with robust standard errors to estimate adjusted relative risks (RRs) and their associated 95% confidence intervals (CIs). Three distinct models were developed to account for potential confounding factors: Model 1 adjusted solely for the calendar year of birth; Model 2 incorporated adjustments for both the calendar year of birth and family characteristics, including mother's age, father's age, mother's BMI, father's BMI, higher education of mother or father, occupation, family structure, maternal complications during pregnancy; and Model 3 encompassed adjustments for the calendar year of birth, family characteristics and child‐specific attributes, including child's age, sex, eyesight, right‐handedness, breast feeding longer than 6 months, birth weight, delivery mode, parity, NICU admission, other developmental disorders and psychiatric medication.

We conducted subgroup analyses and visually presented the comparisons between groups, such as obesity, overweight/obesity and thinness, by stratifying the data according to child age, sex, delivery mode, breast feeding status and family structure, adjusting for the covariates included in Model 3. To evaluate potential interactions between postterm birth and subgroup variables, we calculated relative excess risk due to interaction (RERI). The results of these analyses were presented as RRs with 95% CIs. The statistical analyses were performed utilising R Statistical Software version 4.2.2.

### 2.6 Missing data

There were no instances of missing data within the primary exposure and outcome variables. However, some covariates displayed minor amounts of missing data: mother's age (*n* = 2172; 1.3%), father's age (*n* = 2044; 1.2%), mother's BMI (*n* = 868; 0.5%), father's BMI (*n* = 641; 0.4%), children's age (*n* = 1428; 0.8%), breast feeding longer than 6 months (*n* = 1092; 0.6%) and birth weight (*n* = 1988; 1.2%). To address these gaps, we employed multiple imputation by chained equations, generating 50 imputed datasets. This method enhances the robustness of our analysis, allowing us to maintain the integrity of our dataset while assessing the impact of these covariates on our findings.

### 2.7 Sensitivity analysis

To ensure the robustness and reliability of our findings, we implemented several sensitivity analyses. First, we compared postterm births to various categories of term births. These included earlyterm births (defined as 37 0/7–38 6/7 weeks of gestation), fullterm births (defined as 39 0/7–40 6/7 weeks of gestation) and lateterm births (defined as 41 0/7–41 6/7 weeks of gestation), based on definitions from previous research.^28^ Second, we utilised both the International Obesity Task Force (IOTF) criteria and Chinese reference data to define childhood overweight and obesity, providing age‐and sex‐specific cutoff points for children aged 2–18 years.^29^, ^30^ Additionally, we defined childhood thinness using a BMI‐for‐age *z*‐score threshold of <−2, aligning with grade 2 thinness in adults. These analyses were conducted to test the consistency and stability of our study results across different classification criteria.

### 2.8 Ethics approval

Ethical approval for the study was obtained from the Ethics Committee of the Shanghai First Maternity and Infant Hospital (KS18156). Parents provided online written consent for their participation in the study.

## 3 RESULTS

### 3.1 Characteristics of the study population

We included 141,002 mother–child pairs, of whom 7314 (5.2%) children were classified as postterm births, characterised by higher birthweight and more likely from nuclear families. Parents of postterm births showed a lower percentage of higher education. For a comprehensive overview of the characteristics of the study subjects, please refer to Table 1.

### 3.2 Associations of term birth, postterm birth and adverse growth outcomes

**FIGURE 2:**
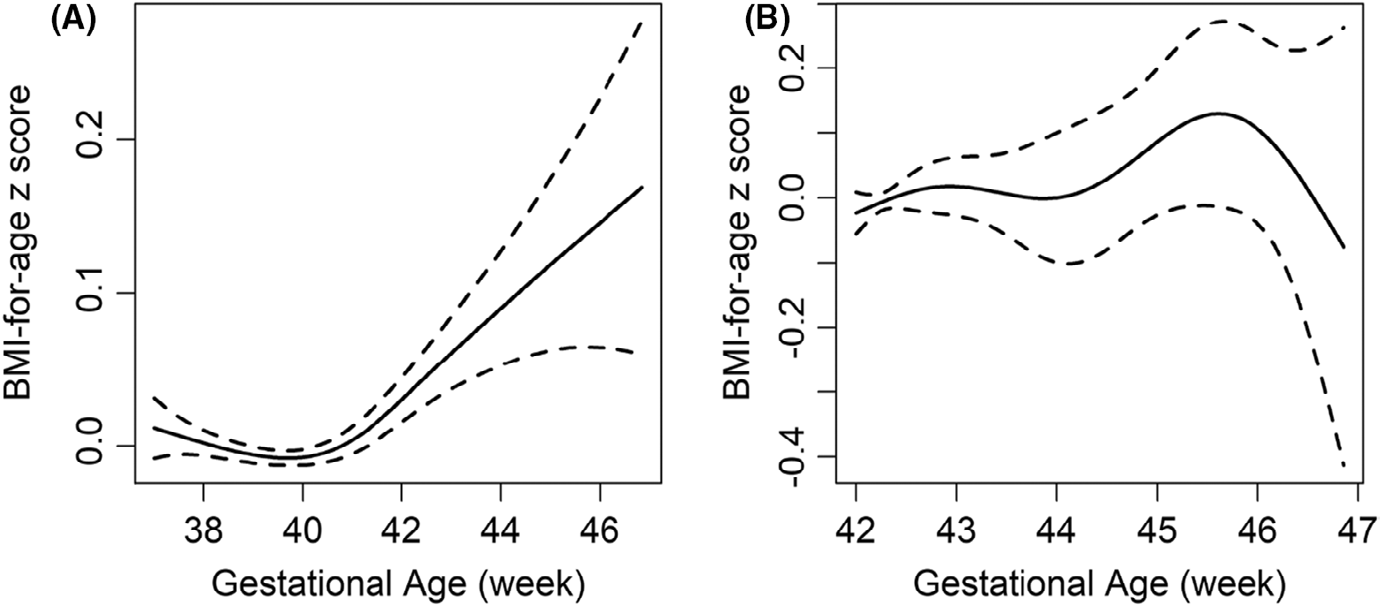
The non‐linear relationship between gestational age and BMI‐for‐age *z* score. (A) Comprises both term and postterm cases (gestational age ≥37 and <47 weeks), whereas (B) exclusively consists of postterm cases (gestational age ≥42 and <47 weeks).

We identified a U‐shaped relationship between gestational age and BMI‐for‐age *z*‐scores, where both lower and higher gestational ages were associated with increased *z*‐scores compared to normal gestational age (Figure 2A). Specifically, among postterm births, BMI‐for‐age *z*‐scores increased with gestational age, peaking at approximately 45.5 weeks, before subsequently decreasing (Figure 2B).

Furthermore, our study showed that children born postterm had a 46% increased risk of obesity, a 27% increased risk of overweight/obesity and a 13% increased risk of thinness, after adjusting for the calendar year of birth, family and child characteristics in Model 3. Similar results were observed in Model 1 and 2 (Table 2).

**TABLE 2:** Associations between postterm pregnancy and risk of adverse growth outcomes.

Outcomes	*n* Events/total	RR (95% CI)		
		Model 1^a^	Model 2^b^	Model 3^c^
Obesity				
Term birth	3186/112,674	1.00 (Reference)	1.00 (Reference)	1.00 (Reference)
Postterm birth	265/5960	1.56 (1.38, 1.76)	1.45 (1.28, 1.64)	1.46 (1.29, 1.65)
Overweight/obesity				
Term birth	9471/118,959	1.00 (Reference)	1.00 (Reference)	1.00 (Reference)
Postterm birth	700/6395	1.37 (1.27, 1.47)	1.29 (1.20, 1.39)	1.27 (1.18, 1.36)
Thinness				
Term birth	14,729/124,217	1.00 (Reference)	1.00 (Reference)	1.00 (Reference)
Postterm birth	919/6614	1.16 (1.09, 1.24)	1.12 (1.06, 1.20)	1.13 (1.06, 1.20)

^a^ Model 1 was adjusted for calendar year of birth only.

^b^ Model 2 was adjusted for calendar year of birth and family characteristics including mother's age, father's age, mother's BMI, father's BMI, higher education of mother, higher education of father, mother's occupation, father's occupation, family structure and maternal complications during pregnancy.

^c^ Model 3 was adjusted for calendar year of birth, family and child characteristics including the child's age, sex, eyesight, right‐handedness, breast feeding longer than 6 months, birth weight, delivery mode, parity, NICU admission, other developmental disorders and psychiatric medication.

### 3.3 Associations of term birth, postterm birth and adverse growth outcomes in different subgroups

**FIGURE 3:**
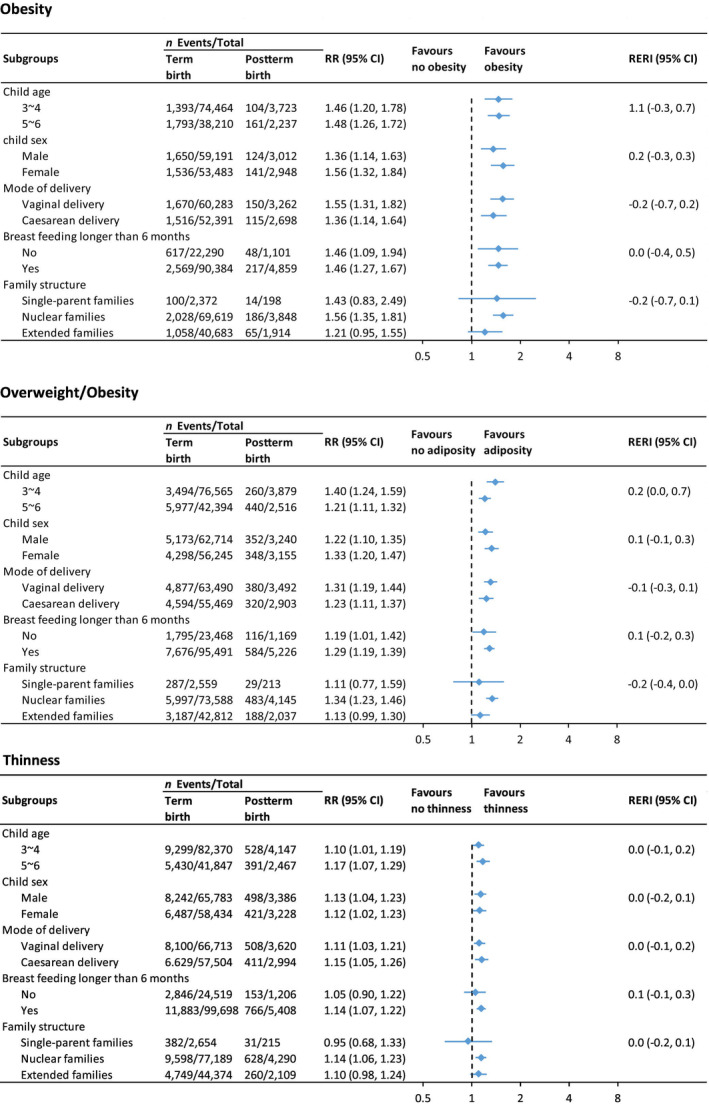
Subgroup analysis of association between postterm birth and risk of adverse growth outcomes.

Figure 3 shows the results of subgroup analyses investigating the association between postterm birth and the risk of adverse growth outcomes. Associations were consistently found in most subgroup analyses. It is noteworthy that the association between postterm birth and obesity was more prominent in children from nuclear families, while the risk estimates in children from single‐parent families and extended families were lower. Similar relative risks were observed for overweight/obesity and thinness across different subgroups.

### 3.4 Sensitivity analysis

Similar associations were found when comparing postterm birth with three categories of term birth (early, full and late) (Table S1). Furthermore, defining overweight and obesity by the IOTF and Chinese reference data and defining thinness as BMI‐for‐age *z* score <−2 did not alter the risk estimates substantially (Table S2).

## 4 COMMENT

### 4.1 Principal findings

In this national population‐based birth cohort study, we discovered that postterm birth, defined as a gestational age of 42 weeks or more, was associated with a higher risk of adverse growth outcomes, including obesity, overweight, and thinness in children aged 3–6 years, after adjusting for a wide range of confounders. Compared to children born at term, those born postterm had higher BMI‐for‐age *z* scores. These findings persisted across various subgroups, indicating a robust association irrespective of demographic or contextual variations. These findings underscore the complex relationship between extended gestational age and childhood growth patterns, highlighting the need for heightened surveillance for children born postterm.

### 4.2 Strengths of the study

Our study offers several strengths that enhance the validity and reliability of our findings. We utilised a large population‐based cohort, which allowed for robust statistical analyses and generalisability of the results. The comprehensive documentation of exposures, variables and outcomes ensured a thorough assessment of the factors under investigation. The study also accounted for various potential confounding variables, thereby strengthening the internal validity of our findings. One of the notable contributions of our study is the identification of an association between postterm birth and the incidence of thinness, overweight and obesity in children. These results not only corroborate existing evidence linking prolonged gestation to higher BMI in children but also shed light on the increased risk of thinness observed in postterm births. This finding highlights the importance of considering the entire spectrum of weight status outcomes when examining the impact of gestational age on childhood health.

### 4.3 Limitations of the data

The study has several limitations. The cross‐sectional design limits the ability to infer causality from the observed associations. Furthermore, relying on parental reports for initial data collection may introduce reporting biases, particularly for historical data like gestational age and early infant feeding practices. The study also did not account for potential regional variations in health practices and access to care, which might influence growth outcomes.

### 4.4 Interpretation

Previous studies investigating the association between postterm birth and childhood obesity have yielded limited and inconsistent results.^22^, ^23^, ^24^ Using a large population sample and controlling a wide range of confounders, our findings identified a positive relationship between postterm birth and the risk of childhood overweight, obesity and thinness. In our study, children born postterm exhibited higher BMI‐for‐age *z*‐scores compared to their term counterparts, consistent with previous research indicating a higher propensity for weight‐related issues in this group. This association persisted even after adjusting for a comprehensive set of confounders, highlighting the robustness of the relationship between prolonged gestational age and subsequent growth outcomes. However, a previous study reported a lower risk of overweight in preschool‐age children born postterm,^24^ which contradicts our results. Nonetheless, our findings are in line with theirs regarding the increased risk of thinness associated with postterm birth. It is worth noting that metabolic similarities have been observed between infants born small‐for‐gestational age (SGA) and those born postterm, providing an explanation for the likelihood of thinness, although this association is typically observed at birth and not throughout infancy.^31^

The elevated risk for both overweight and thinness in postterm children suggests a complex interaction between gestational age and metabolic regulation. This dual risk pattern may reflect a unique metabolic programming that occurs when birth extends beyond the typical gestational period. Some infants may develop a more efficient fat storage system, predisposing them to obesity,^22^ while others might have altered energy expenditure rates, leading to thinness.^24^ The underlying causes of metabolic changes in postterm births are still unclear but may be attributed to genetic factors or a suboptimal foetal environment during late gestation.^32^, ^33^ Several studies have hypothesised that metabolic disruptions in postterm infants could stem from hormonal and nutritional imbalances that arise as the placenta ages and its function declines, potentially altering nutrient transfer to the fetus.^24^, ^34^ Prolonged gestation exposes postterm newborns to physiological stress,^35^ however, significant undernutrition during this period is unlikely compared to preterm births.^36^ Therefore, negative effects may not be directly linked to the extended in utero period itself. Further research is needed to explore metabolic alterations in postterm pregnancies and their relationship with childhood thinness, as existing studies present inconsistent findings. Another hypothesis suggests that inadequate gestational weight gain prior to pregnancy increases the likelihood of having thin children.^37^ Consequently, further research is needed to explore the biological pathways involved and to determine effective strategies for intervention and support for this vulnerable group.

The association between postterm and obesity, overweight and thinness is not different between sex. The existing literature provided inconsistent findings regarding the sex‐specific effects of postterm births on child development. Particularly, a prior study reported significant sex‐dependent associations with obesity, suggesting a potential sex difference in susceptibility to the stress associated with prolonged gestation.^22^ However, the interpretation of this study was significantly constrained by a small sample size (*n* = 525, including only 17 boys and 20 girls born postterm) and a lack of adjustment for confounding factors. The current study's key findings, on the other hand, indicated a sex‐independent relationship. The specific mechanisms underlying these observed sex differences are not well‐established in the existing literature, warranting further research for clarification.

Nutritional practices, particularly breast feeding, significantly impact children's growth and development post‐birth. Our study found that prolonged breast feeding (beyond 6 months) correlates with lower risks of overweight, obesity and thinness in children born postterm, supporting WHO recommendations to breastfeed for 6 months to mitigate future obesity risks.^38^, ^39^, ^40^ Breast milk contains hormones such as leptin, adiponectin and ghrelin, which play crucial roles in adipose tissue regulation and may protect against obesity.^38^ In contrast, studies in diverse ethnic groups, including Brazilian and Hispanic children, have shown that the protective effects of breast feeding on BMI may diminish after the first year due to dietary and lifestyle changes.^41^, ^42^ Further research in a Chinese province suggested that breast feeding duration influences obesity and thinness differently depending on whether infants are born preterm or postterm, though these findings are not generalisable across China.^43^ Similarly, a Thai study indicated that extended breast feeding correlates with increased skinfold thickness and fat mass, suggesting that longer breast feeding duration might not uniformly prevent obesity.^44^ Contrasting studies in East Asian populations have linked shorter breast feeding durations with higher BMI and increased cardiometabolic risks.^45^, ^46^ These discrepancies highlight the complexity of breast feeding's impact on childhood growth outcomes, influenced by factors such as breast feeding duration and child age. Future research should explore these variables to better understand and optimise breast feeding recommendations, particularly for different gestational ages.

The family environment, particularly in nuclear families which include parents and their children, significantly influences children's health behaviours and obesity risks.^47^, ^48^, ^49^, ^50^ Our study found that postterm births are associated with higher obesity risks in nuclear families, possibly due to higher incomes that allow for greater food consumption.^51^ However, this trend does not extend to single‐parent or extended families, nor does it appear in non‐breastfed children, suggesting that other factors like sibling presence and parental education may not uniformly affect dietary habits, as evidenced by varied impacts in different socioeconomic settings.^52^, ^53^, ^54^, ^55^, ^56^

## 5 CONCLUSIONS

In this population‐based birth cohort study, we observed an increased risk of childhood obesity, overweight and thinness associated with postterm birth in children aged 3–6 years, independent of sex. These findings highlight the potential long‐term consequences of prolonged gestation on the adverse weight‐related outcomes of children. Our results emphasise the importance of identifying and addressing the predictors of postterm birth to minimise the negative health effects on the offspring.

## AUTHOR CONTRIBUTIONS

The corresponding authors (Du and Hua) had full access to all of the data in the study and took responsibility for the integrity of the data and the accuracy of the data analysis. Concept and design: Hua, Du. Acquisition, analysis or interpretation of data: Zheng, You, Su. Drafting of the manuscript: Ahmad Suhaimi, Zheng, Du. Critical revision of the manuscript for important intellectual content: Hua, Du, Williams. Statistical analysis: Zheng, You. Obtained funding: Hua, Du. Administrative, technical or material support: You. Supervision: Hua, Du.

## FUNDING INFORMATION

This study was supported by the National Natural Science Foundation of China (81673179), the Science and Technology Commission of Shanghai Municipality (21DZ2202000), Shanghai Municipal Health Commission (2020YJZX0213), Pudong Municipal Health Commission (PW2020D‐11).

## CONFLICT OF INTEREST STATEMENT

The authors declare no conflict of interest.

## Supporting information


Table S1.
Table S2.

## Data Availability

The datasets analysed in the current study are available from the corresponding authors on reasonable request.
