# Supplementary material for: Association between postterm birth and adverse growth outcomes in children aged 3–6 years: A national retrospective cohort study
Source: Paediatr Perinat Epidemiol. 2024 Sep 19;39(1):30–40. doi: 10.1111/ppe.13122 (PMC11781517; doi:10.1111/ppe.13122)
Supplement: Supplementary file 1 — Table S1. Table S2. [file PPE-39-30-s001.docx]

# eTable 1. Associations between postterm birth and risk of adverse growth outcomes in preschool-aged children-sensitivity analyses comparing postterm birth versus early, full, and late birth.

|  | **Obesity** | | **Overweight/Obesity** | | **Thinness** | |
| --- | --- | --- | --- | --- | --- | --- |
| **Reference category** | **N events/N total** | **RR (95% CI)** | **N events/N total** | **RR (95% CI)** | **N events/N total** | **RR (95% CI)** |
| Comparison 1 |  |  |  |  |  |  |
| Earlyterm birth | 1,091/35,478 | 1.00 (Reference) | 3,259/37,646 | 1.00 (Reference) | 4,954/39,341 | 1.00 (Reference) |
| Postterm birth | 265/5,960 | 1.40 (1.23, 1.60) | 700/6,395 | 1.20 (1.11, 1.30) | 919/6,614 | 1.09 (1.02, 1.17) |
| Comparison 2 |  |  |  |  |  |  |
| Fullterm birth | 1,875/69,039 | 1.00 (Reference) | 5,601/72,765 | 1.00 (Reference) | 8,790/75,954 | 1.00 (Reference) |
| Postterm birth | 265/5,960 | 1.49 (1.31, 1.69) | 700/6,395 | 1.30 (1.21, 1.40) | 919/6,614 | 1.14 (1.07, 1.22) |
| Comparison 3 |  |  |  |  |  |  |
| Lateterm birth | 220/8,157 | 1.00 (Reference) | 611/8,548 | 1.00 (Reference) | 985/8,922 | 1.00 (Reference) |
| Postterm birth | 265/5,960 | 1.46 (1.23, 1.74) | 700/6,395 | 1.37 (1.24, 1.53) | 919/6,614 | 1.16 (1.06, 1.26) |

# eTable 2. Associations between postterm birth and risk of adverse growth outcomes in preschool-aged children-sensitivity analysis using the Chinese and the IOTF criteria to define childhood obesity, and using BMI for age <-2 to define childhood thinness

|  | **Obesity** | | **Overweight/obesity** | | **Thinness** | |
| --- | --- | --- | --- | --- | --- | --- |
| **Different criteria** | **N events/N total** | **RR (95% CI)** | **N events/N total** | **RR (95% CI)** | **N events/N total** | **RR (95% CI)** |
| IOTF criteria |  |  |  |  |  |  |
| Term birth | 8,441/118,806 | 1.00 (Reference) | 21,898/132,263 | 1.00 (Reference) | 1,425/111,790 | 1.00 (Reference) |
| Postterm birth | 644/6,425 | 1.36 (1.26, 1.47) | 1,424/7,205 | 1.16 (1.10, 1.21) | 109/5,890 | 1.30 (1.08, 1.58) |
| Chinese criteria |  |  |  |  |  |  |
| Term birth | 20,040/118,444 | 1.00 (Reference) | 33,859/132,263 | 1.00 (Reference) | 1,425/99,829 | 1.00 (Reference) |
| Postterm birth | 1,318/6,426 | 1.18 (1.12, 1.24) | 2,097/7,205 | 1.11 (1.07, 1.15) | 109/5,217 | 1.31 (1.08, 1.59) |
